# Association of metabotropic glutamate receptor 7 gene with nicotine dependence in a Chinese Han population: A cross-sectional candidate-gene association study

**DOI:** 10.18332/tid/219820

**Published:** 2026-08-01

**Authors:** Jinjie Zhao, Mengying Li, Ruibo Wang, Wenjing Shen, Meng Li

**Affiliations:** 1 Faculty of Open Education, Yunnan Open University, Kunming, China; 2 Yunnan Academy of Tobacco Science, Kunming, China

**Keywords:** extended haplotype homozygosity, neuropsychiatric disorders, replication analysis, tobacco addiction

## Abstract

**INTRODUCTION:**

Despite a global decline in prevalence, cigarette smoking remains a significant public health issue. Tobacco addiction is principally caused by nicotine dependence (ND). This study aimed to investigate the relationship between the metabotropic glutamate receptor 7 (*GRM7*) gene and ND in Chinese adults.

**METHODS:**

This two-stage, cross-sectional, candidate-gene association study first enrolled a discovery cohort of 1609 participants from Kunming, Yunnan, China (2017–2019), followed by a replication stage involving 1329 individuals from Jincheng and Taiyuan, Shanxi, China (2012–2013). Association analysis between the *GRM7* gene and ND was performed at individual SNP, haplotype, and whole-gene levels. Logistic and linear regression models were employed for single-SNP association analyses. Heaviness of smoking index (HSI) and Fagerström test for nicotine dependence (FTND) were used to assess ND.

**RESULTS:**

Rs77452956, rs74791991, and rs2176404 were significantly associated with ND proxied by HSI, with p-values of <0.001, 0.001, and 0.002, respectively. In the replication analysis, rs74791991 and rs2176404 showed associations with ND (p=0.006 and 0.082, respectively), while rs77452956 was linked to smoking status (p=0.014). Haplotype-based association analysis revealed that two haplotypes, C-C-T and T-G-C, comprising rs74791991, rs111848366, and rs7633313, were associated significantly with HSI, with p<0.001 and p=0.003, respectively. Gene-based analysis also revealed a significant association between *GRM7* and HSI (p=0.006). Functional assessment suggested that rs77452956 and rs74791991 have regulatory capacity. Furthermore, the derived C alleles of rs74791991, rs2176404, and rs77452956 showed accelerated evolution.

**CONCLUSIONS:**

We identified three SNPs, rs74791991, rs2176404, and rs77452956, associated with ND in Han Chinese individuals. Our findings suggest a potential role of *GRM7* in susceptibility to smoking-related behaviors and warrant further investigation to determine its relevance in smoking cessation.

## INTRODUCTION

Although the number of tobacco users has declined steadily over the past two decades, an estimated 1.2 billion people worldwide still used tobacco in 2024^1^. China ranks as the world’s foremost producer and consumer of tobacco products, with approximately 25.6% of adults, aged ≥18 years, smoking^2^. Nicotine dependence (ND) is widely recognized as a key factor in persistent smoking^3^. Understanding the neurobiological mechanisms underlying ND is crucial for developing effective smoking cessation interventions.

Metabotropic glutamate receptor 7 (*GRM7*, also known as *mGlu*7) is a member of the group III metabotropic glutamate receptor family. Expression of *GRM7* is widespread throughout the central nervous system (CNS), e.g. the prefrontal cortex, hippocampus, and amygdala^4^. *GRM7* is thought to be essential for a wide range of CNS functions, including memory, learning, cognition, and emotion^5^. Notably, *GRM7* was found to be involved in multiple neurodevelopmental and neuropsychiatric disorders, including but not limited to autism^6^, bipolar disorder^7^, and schizophrenia^8^. For example, in valproic acid-treated C57BL/6J mice, overexpression of *GRM7* in the prefrontal cortex could ameliorate autism spectrum disorder behaviors^6^.

It has been reported that down-regulation of *GRM7* expression enhanced ethanol consumption and conditioned place preference in male rats^9^. Activation of *GRM7* inhibits cocaine-seeking, reducing drug self-administration in rats^10^. Additionally, *GRM7* was linked to vulnerabilities to ND in a 520000 single-nucleotide polymorphisms (SNPs)-based genome-wide association study (GWAS)^11^. Another GWAS further supported the involvement of *GRM7* in smoking initiation and current smoking, in which four SNPs, i.e. rs162773, rs13070476, rs779714, and rs1868616, were found to be associated^12^. In a COPDGene cohort, a polymorphic CNV segment from the intergenic region of *GRM7* showed a significant association with pack-years (smoked cigarette packs per day times the years of smoking)^13^. These findings highlight the intricate role of *GRM7* in addiction neurobiology.

Deciphering differences in the evolving pattern of ND associated genetic variants between populations could facilitate the development of personalized medicine approaches, enabling more effective and individualized treatments for addiction by considering genetic backgrounds. It may also help understand how the addiction behavior developed and evolved during the human self-domestication history. The present study aimed to systematically investigate the association between *GRM7* and ND, and to explore the biological functions of the identified susceptibility loci. We further characterized the molecular evolution of these loci to better address their evolving pattern in different populations.

## METHODS

### Study design and participants

This is a cross-sectional candidate-gene association study. The discovery stage of this study included 1609 participants (1014 smokers and 595 non-smokers) from Yunnan, China. Of these, 1184 were derived from an earlier cohort, while the remaining 425 were collected using the same methodology^14^. The recruitment period was from September 2017 to August 2019. The samples used for replication analysis were enrolled in a previous study (2012–2013; n=1329; 805 smokers and 524 non-smokers)^14^. All participants signed a written informed consent form to participate in this study.

This study was approved by the institutional review board (the Biomedical Ethics Committee of the Joint Institute of Tobacco and Health) on human studies (No. JITH201801, November, 2018).

### Phenotype assessment

To ensure trustworthy results, only participants who had smoked at least 100 cigarettes in their lifetimes were classified as smokers. In total, the discovery sample consisted of 1014 current smokers, 67 former smokers, and 528 never smokers. ND was measured by using the HSI derived from two items: time to the first cigarette of the day and number of cigarettes smoked per day. The total HSI score ranges from 0 to 6, with higher scores indicating greater ND. In the discovery stage, heavy smokers (HSI ≥3) were defined as the case group (n=398). Light smokers (HSI <3) were treated as the control group (n=616). In the replication stage, the Fagerström test for nicotine dependence (FTND) was employed to quantify the severity of ND. The total FTND score ranges from 0 to 10, with higher scores representing increased levels of dependence. These scores were treated as a continuous variable for regression analysis.

### Genotyping and SNP selection

Sequencing and genotyping were performed as outlined in Li et al.^14^. To obtain reliable genotyping results, variants were further filtered by stringent criteria, using the Genome Analysis Toolkit^15^. Hard filtering criteria included: quality by depth (QD) ≥2.0, mapping quality (MQ) ≥40.0, strand odds ratio (SOR) <3.0, Fisher strand (FS) <60.0, mapping quality rank sum test (MQRankSum) < -12.5, and read position rank sum test (ReadPosRankSum) < -8.0. SNPs that met these criteria were retained, while those failing any of the thresholds were excluded.

To select tag SNPs, a preliminary GWAS and linkage disequilibrium (LD) analysis were conducted. The GWAS showed that no SNPs of the *GRM7* were significantly associated with ND (p >5×10^-8^). In the LD analysis, SNPs with an r^2^ ≥0.8 were considered to be in high LD. Within each LD block, the SNP with the lowest p*-*value was designated as the tag SNP, and other SNPs were excluded. Additionally, SNPs with p>0.05 were filtered out.

The above steps resulted in a final set of 25 tag SNPs for the *GRM7* gene, i.e. rs77452956, rs1829728, rs6443144, rs111848366, rs74791991, rs7639899, rs7633313, rs9850138, rs75750300, rs17043965, rs62245596, rs2176404, rs1553373, rs62245333, rs2323590, rs58637544, rs141802097, rs6795860, rs2324442, rs9840406, rs2021128, rs62245597, rs3828431, rs9311977, and rs2572687. All of them had a genotyping call rate >88%, a minor allele frequency (MAF) >0.01, and a Hardy-Weinberg equilibrium (HWE) p>0.0001.

### Statistical analysis

Individual SNP-based association tests were conducted using PLINK (v.1.9). Logistic regression was employed for categorical outcomes (HSI and smoking status), while linear regression was used for the quantitative FTND score under an additive genetic model. All statistical models were adjusted for covariates, including sex, age, and the first three principal components 1, 2, and 3 (PC1, PC2, PC3) derived from a principal component analysis (PCA). In the discovery phase, we applied a Bonferroni-corrected p=0.002 (0.05/25) to account for multiple testing. For the subsequent replication stage, results were considered statistically significant at a nominal level of p<0.05.

To apply haplotype-based association analysis, pairwise linkage disequilibrium and haplotype blocks of the selected SNPs were calculated by the *Haploview* software (v.4.2)^16^. The Haplo Stats (v1.7.9) R package was adopted to perform haplotype-based association analysis^17^. The significance level was adjusted using Bonferroni correction. All statistical analyses involving R were implemented in the R software environment (v4.1.1)^18^.

Gene-based association tests were carried out by using MAGMA^19^. The multiple linear principal components regression model was used, and the parameter ‘multi’ was specified as ‘all’. Gene-based association analyses were performed in two rounds: one with covariates and one without. In the analysis that included covariates, sex, age, and the first three principal components (PC1, PC2, and PC3) were included to account for potential confounding factors.

### *In silico* functional prediction

Variants were annotated using the RegulomeDB^20^ and Genome Wide Annotation of Variants^21^. Tissue-specific gene expression results were obtained from the GTEx project^22^.

### Extended haplotype homozygosity (EHH) analysis

The extended haplotype homozygosity was analyzed by using the *rehh* (v3.2.2) R package^23^. All the SNPs of the *GRM7* that passed the quality control criteria were included. Genotype data for African (AFR), Ad Mixed American (AMR), East Asian (EAS), European (EUR), and South Asian (SAS) populations were retrieved from the 1000 Genomes Project^24^. The Han Chinese (CHB) genotype data were isolated and merged with our own genotype data, while the remaining population genotype data were categorized as non-Chinese.

## RESULTS

### Individual SNP-based association analysis of *GRM7* with ND in the Chinese Han population

Individual SNP-based association analysis of ND was performed with 398 heavy smokers versus 616 light smokers. Significant associations with HSI were observed (Table 1). Rs77452956 exhibited the strongest association (p<0.001). After Bonferroni correction for multiple testing, 12 SNPs remained significant with p*<*0.05 (i.e. rs77452956, rs1829728, rs6443144, rs111848366, rs74791991, rs7639899, rs7633313, rs9850138, rs75750300, rs17043965, rs62245596, and rs2176404) (Table 1). The most significantly associated rs77452956 has an odds ratio of 0.683 and a minor allele frequency of 0.301.

**Table 1:** Individual SNP association analysis with HSI (heavy vs light smokers), a cross-sectional study in Yunnan, China, 2017–2019 (N=1014)

*SNP*	*Location*	*p*	*OR*	*Minor/major allele*	*MAF*	*Type*
**rs77452956**	chr3_5935087	<0.001	0.683	C/A	0.301	intergenic
**rs1829728**	chr3_6687178	<0.001	1.457	C/T	0.292	intronic
**rs6443144**	chr3_7983344	<0.001	1.518	A/C	0.218	intergenic
**rs111848366**	chr3_5940312	0.001	0.664	A/G	0.227	intergenic
**rs74791991**	chr3_5936350	0.001	0.659	C/G	0.220	intergenic
**rs7639899**	chr3_6681225	0.001	0.707	C/G	0.355	intronic
**rs7633313**	chr3_5943965	0.001	0.682	C/G	0.263	intergenic
**rs9850138**	chr3_6680311	0.001	1.440	T/C	0.291	intronic
**rs75750300**	chr3_5791207	0.001	0.566	G/A	0.103	intergenic
**rs17043965**	chr3_5990857	0.001	0.668	G/T	0.232	intergenic
**rs62245596**	chr3_5782993	0.002	0.624	C/A	0.143	intergenic
**rs2176404**	chr3_5947663	0.002	0.676	C/T	0.230	intergenic
rs1553373	chr3_6687502	0.002	0.720	C/A	0.356	intronic
rs62245333	chr3_6709768	0.004	0.724	T/C	0.315	intronic
rs2323590	chr3_6097045	0.004	1.335	T/A	0.467	intergenic
rs58637544	chr3_6714091	0.004	0.727	A/G	0.317	intronic
rs141802097	chr3_5423493	0.005	0.692	T/C	0.196	intergenic
rs6795860	chr3_5994754	0.006	0.707	G/A	0.234	intergenic
rs2324442	chr3_7977880	0.006	2.812	C/A	0.018	intergenic
rs9840406	chr3_7138036	0.007	0.756	T/A	0.410	intronic
rs2021128	chr3_6703833	0.007	0.752	G/C	0.357	intronic
rs62245597	chr3_5785568	0.008	0.618	C/T	0.099	intergenic
rs3828431	chr3_7505350	0.008	0.602	T/A	0.089	intronic
rs9311977	chr3_7128113	0.009	0.764	C/A	0.416	intronic
rs2572687	chr3_5901084	0.009	1.479	G/A	0.133	intergenic

MAF: minor allele frequency. Bonferroni corrected significant SNPs are highlighted in bold. HSI: Heaviness of smoking index (Score 0–6). Heavy smokers were defined as HSI ≥3; light smokers as HSI <3.

### Replication analysis

Replication of the 12 SNPs was carried out in a cohort consisting of 805 smokers. One SNP (rs74791991) was significant (p=0.006) and another SNP (rs2176404) was marginally significant (p=0.082), in association with FTND, under an additive genetic model using linear regression (Table 2). In addition, rs77452956 showed an association with smoking status (805 smokers vs 524 non-smokers, p=0.014).

**Table 2:** Individual SNP association analysis with smoking behavior traits, a cross-sectional replication study in Shanxi, China, 2012–2013 (N=1329)

*Phenotype*	*SNP*	*Minor/ major allele*	*Location*	*β*	*SE*	*STAT*	*p*
**FTND**	rs74791991	C/G	chr3_5936350	0.207	0.075	2.780	0.006
	rs2176404	C/T	chr3_5947663	0.125	0.072	1.741	0.082
**Smoking status**	rs77452956	C/A	chr3_5935087	-0.206	0.083	-2.462	0.014

β: regression coefficient. SE: standard error. STAT: coefficient t-statistic. FTND: Fagerström test for nicotine dependence.

### Haplotype-based association analysis of GRM7 with ND in the Chinese Han population

**Figure 1:**
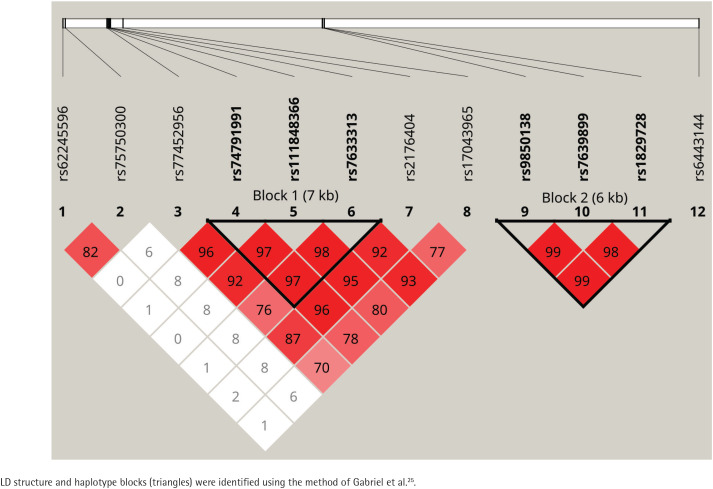
The linkage disequilibrium plot depicts the distribution of GRM7 12 SNPs and their corresponding haplotype blocks in the Chinese Han samples collected in Kunming, Yunnan (2017–2019; N=1609). Blocks represent genomic segments with minimal historical recombination, where the normalized linkage disequilibrium coefficient for most SNP pairs (at least 95%) shows a 95% confidence interval between 0.70 and 0.98

Among the 12 SNPs associated with ND in the discovery sample, two LD blocks (D’ >0.97) were determined according to the haplotype block definition of Gabriel et al.^25^ (Figure 1). Haplotype-based association analysis was then carried out using these two haplotype blocks. Two haplotypes, C-A-C and G-G-G, formed by rs9850138, rs7639899, and rs1829728, showed significant associations with HSI. The haplotype score was -3.289 (p=0.001) and 3.437 (p<0.001), respectively (Table 3). Further, we found two other haplotypes, C-C-T and T-G-C, constituted by rs74791991, rs111848366, and rs7633313, to be associated with HSI (haplotype score -3.309, p<0.001; haplotype score 2.931, p=0.003, respectively) (Table 3).

**Table 3:** Haplotypes of the GRM7 gene associated with HSI (heavy vs light smokers) in the discovery sample (N=1014)

*SNP combination*	*Haplotype*	*Haplotype frequency*	*Haplotype score*	*p-Hap*	*p-Global*
rs9850138 rs7639899 rs1829728	C-A-C	0.208	-3.289	**0.001**	0.026
	C-G-G	0.003	-1.590	0.112	
	G-G-C	0.034	-0.584	0.559	
	G-A-G	0.002	-0.339	0.735	
	G-A-C	0.008	0.349	0.727	
	G-G-G	0.742	3.437	**<0.001**	
rs74791991 rs111848366 rs7633313	C-C-T	0.362	-3.309	**<0.001**	0.008
	C-G-T	0.346	0.443	0.658	
	C-G-C	0.002	0.884	0.377	
	T-G-C	0.287	2.931	**0.003**	

p-Hap: Haplotype-specific p-value associated with a specific haplotype’s association with the trait. Significant p-Hap values after Bonferroni correction are shown in boldface. p-Global: Global p-value associated with the overall test of all haplotypes in the model. A significant p-Global indicates that at least one haplotype is associated with the trait.

### Gene-based association analysis

The MAGMA gene-based association test was performed on all the SNPs of the *GRM7* gene. In the analysis without covariates, *GRM7* was significantly associated with HSI (p=0.006), with a standardized gene-level association score of 2.52 (Table 4). However, this association became non-significant when sex, age, and the first three principal components (PC1, PC2, and PC3) were included as covariates (p=0.749).

**Table 4:** Gene-based association analysis of GRM7 genetic variants with HSI (heavy vs light smokers) in the discovery sample (N=1014)

*Model*	*Start position (bp)*	*Stop position (bp)*	*ZSTAT*	*p*
Multiple linear principal components regression model (without covariates)	6902802	7783218	2.520	0.006
Multiple linear principal components regression model (covariates: sex, age, and the first three principal components)			-0.671	0.749

ZSTAT: standardized gene-level association score, derived from the aggregation of individual SNP p-values within the gene. Higher ZSTAT values indicate stronger evidence of association.

### Functional assessment

Rs77452956 had a RegulomeDB probability score of 0.3, showing a regulatory potential. According to RegulomeDB prediction results, motifs altered by rs77452956 could bind to the transcription factors developing brain homeobox 1 (DBX1), forkhead box proteins (e.g. FOXA1, FOXC1, FOXF1, FOXJ1, and FOXO3), and POU class 3 homeobox 1 (POU3F1). Rs74791991 might alter nuclear receptor subfamily 3, group C, member 1 and 2 (NR3C1 and NR3C2) binding motifs, with a RegulomeDB probability score of 0.6. For rs2176404, the RegulomeDB probability score was 0.18, and no position-weight matrix for TF binding data was reported. Using the GWAVA tool, rs74791991 was found to fall in a predicted transcribed region. Rs77452956, rs74791991, and rs2176404 were located in repressed segments in 6, 5, and 6 cell lines, respectively. In the brain, the most abundant chromatin state of these three SNP regions was the quiescent state, with low levels of all histone marks and high DNA methylation. In a ChIP-seq experiment on human HEK293 cells, the region of rs74791991 exhibited high binding ability to the *SCRT2* (Scratch Family Transcriptional Repressor 2) gene (Supplementary file Figure S1). In the GTEx project, no association data were found for rs77452956, rs74791991, and rs2176404. While for rs7639899, rs1829728, and rs9850138, which formed haplotypes closely associated with HSI, significant associations with intron-excision ratio were found in splicing quantitative trait loci (sQTL) analysis, with p-values of 1.70×10^-31^, 2.68×10^-8^, and 6.23×10^-6^, respectively (Supplementary file Figure S2). Rs7639899 was also associated with expression quantitative trait loci (eQTL) in the analysis (p=3.81×10^-8^, Supplementary file Figure S3).

### Evolution of rs77452956, rs74791991, and rs2176404 in the Chinese Han population

**Figure 2:**
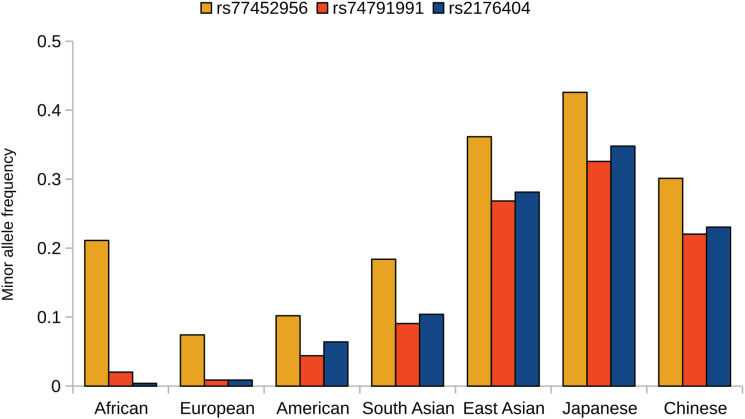
The minor allele frequency distribution of rs77452956, rs74791991, and rs2176404 in African, European, American, South Asian, East Asian (including Japanese and Chinese), Japanese, and Chinese populations, based on data from the 1000 Genomes Project and 1609 Chinese samples obtained in this study. Part of the Chinese data also sourced from the 1000 Genomes Project

For rs77452956, rs74791991, and rs2176404, a higher proportion of the minor alleles was detected in the East Asians, especially in Chinese and Japanese (Figure 2). To gain insights into their evolving patterns, we further performed an extended haplotype homozygosity analysis. As shown in Supplementary file Figure S4, the decay of the rs77452956 C allele was slower than that of its A allele in the Chinese population. Such a phenomenon was not observed in other ethnic groups. This implied that recent positive selection had specifically acted on rs77452956 in the Chinese population. While for rs74791991 and rs2176404, slow decays of the derived alleles (i.e. the C alleles) were observed in both Chinese and non-Chinese ethnic groups (Supplementary file Figure S4). No significant difference was found between Chinese and non-Chinese populations.

## DISCUSSION

This study provides evidence for the involvement of the *GRM7* gene in ND within the Chinese Han population. We identified 12 SNPs significantly associated with ND, with rs74791991, rs2176404, and rs77452956 demonstrating associations that persisted in the replication sample. To date, multi-stage association studies (discovery and replication) exploring the relationship between *GRM7* and ND are underrepresented in the current bibliography. We also found two haplotypes, C-C-T and T-G-C, formed by rs74791991, rs111848366, and rs7633313, significantly associated with HSI. To the best of our knowledge, this is the first report that the *GRM7* gene is associated with ND, both at the single SNP and at the haplotype level. Our findings provide further support for the limited existing evidence linking *GRM7* to smoking behaviors, consistent with the proposed role of *GRM7* in substance dependence^9,10^.

According to RegulomeDB prediction data, the binding targets of rs77452956 include transcription factors involved in neural functions. For example, Dbx1 regulates the specification of hypothalamic nuclei^26^. Pou3f1 facilitates neural differentiation during the transition from epiblast to neural progenitor cells, and activates neural lineage genes, showing neural fate-promoting activity^27^. These results indicated that neuron-related regulation was an important mechanism by which *GRM7* modulates ND. On the other hand, transcription factors interacting with rs77452956 are considered to be associated with smoking-induced physiological changes. For instance, FOXJ1 was required for the development of ciliogenesis in bronchial epithelium^28^. It can promote airway epithelium cilia growth suppressed by cigarette smoke components^29^. In COPD patients, FOXO3 was inactivated by smoking exposure, eliciting an accelerated aging of lung tissues^30^. Besides, RegulomeDB search results suggested that multiple transcription factors involved in cancer were potential targets of rs77452956, e.g. FOXA1, FOXC1, FOXF1, and FOXO3. FOXA1 is crucial in prostate development and in neuroendocrine prostate cancer progression^31^. Based on mechanism and clinical evidence, FOXC1 was perceived as a pivotal biomarker in different cancers, such as lung cancer, breast cancer, and gastrointestinal cancer^32^. For NR3C1 and NR3C2, two putative binding targets of rs74791991, existing evidence demonstrated that they play a role in drug addiction behaviors, such as dependence on cocaine^33^. Importantly, ChIP-seq profiling revealed that rs74791991 could bind to the *SCRT2* gene, a transcription regulator modulating neuron differentiation and migration^34^. These findings indicate that rs74791991 very likely represents a functional SNP and confers high risk susceptibility to ND.

Among the 12 SNPs found to be associated with ND in the discovery sample, rs9850138, rs7639899, and rs1829728 did not achieve statistical significance in the replication analysis. Despite this, two haplotypes, i.e. C-A-C and G-G-G, derived from rs9850138, rs7639899, and rs1829728 showed significant association with HSI. In addition, all three of these SNPs reached statistical significance in sQTL experiments, suggesting that they can affect the alternative splicing of the pre-mRNA of *GRM7*. Moreover, rs7639899 is believed to have the ability to regulate the expression of *GRM7* according to the eQTL analysis. Such results suggest that rs9850138, rs7639899, and rs1829728 may contribute to carrier’s ND by controlling *GRM7* expression at the transcriptional and post-transcriptional levels.

Extra support for the association between *GRM7* and HSI was also found in the gene-based analyses. A significant association with HSI was observed when analyzing the data without covariates, suggesting a potential role of *GRM7* in smoking behavior. This association became non-significant when adjusting for covariates such as sex, age, and principal components, indicating that other factors may confound the relationship. These results also underscore the complexity of genetic influences on smoking behavior and highlight the importance of selecting appropriate covariates in genetic association studies. It is warranted to validate the gene-level associations in further research.

Estimates of EHH revealed recent positive selection signals on rs74791991, rs77452956, and rs2176404, suggesting accelerated evolution of *GRM7* across different populations. This is not surprising given that genes involved in nervous system and brain activities usually evolved at a fast rate during human self-domestication^35^. This observation also aligns with the finding that both rs74791991 and rs77452956 likely play a crucial role in the central nervous system. Of note, rs77452956 was positively selected in the Chinese population but not in other ethnic groups, e.g. Europeans and Africans, which might indicate a population-specific susceptibility during evolutionary history.

### Limitations

There are several limitations to this study. First, the number of subjects included in this study was not sufficiently large to provide adequate statistical power. None of the SNPs in the *GRM7* gene reached the genome-wide significance threshold of 5×10^-8^ in the GWAS on ND. Despite this, single SNP association analysis revealed associations between *GRM7* and ND. More studies with larger and independent samples are needed to validate our identifications. Second, known functional information about the associated loci rs77452956, rs74791991, and rs2176404 is still rare. Although we have adopted several bioinformatics tools to unveil the underlying mechanisms through which these variants influence ND, understanding their biological relevance will continue to be challenging until supporting evidence from molecular experiments becomes available. Third, ND was proxied by FTND in the replication analysis, different from HSI, which was used in the discovery stage. Though both FTND and HSI work well in measuring ND^36^, this may introduce minor discrepancies in the consistency of this work. Finally, even if rs77452956 displays an association with ND, the application of this variant for drug addiction precision targeted therapy is unlikely and not advisable, given that rs77452956 also have a putative role in cancers.

## CONCLUSIONS

Using individual SNP-based, haplotype-based, and gene-based association analyses, we found direct associations between *GRM7* gene and ND in the Chinese Han population, e.g. rs74791991, rs2176404, and rs77452956. Rs74791991 can bind to transcription factors NR3C1 and NR3C2, which are known to contribute to drug addiction traits, while rs77452956 may participate neural activity regulation. Rs74791991, rs2176404, and rs77452956 were found to experience recent positive selection. Further elucidating their biological function will advance our understanding of the genetic determinants of nicotine addiction.

## Supplementary Material



## Data Availability

The original datasets presented in this article are not readily available due to China’s restriction, ‘Regulations on the Management of Human Genetic Resources (HGR Regulations)’. Requests to access the variants data should be directed to the corresponding author.
