# Supplementary material for: Association of metabotropic glutamate receptor 7 gene with nicotine dependence in a Chinese Han population: A cross-sectional candidate-gene association study
Source: Tob Induc Dis. 2026 Aug 1;24:10.18332/tid/219820. doi: 10.18332/tid/219820 (PMC13450126; doi:10.18332/tid/219820)
Supplement: Supplementary file 1 [file TID-24-137-s1.pdf]

Tobacco Induced Diseases

Supplementary file

© 2026 Zhao J. et al.

DOI:
10.18332/tid/219820

The content has been provided by the author(s) and has not been reviewed, verified, or endorsed by European Publishing. It may not have undergone peer review. The views, opinions, and recommendations expressed are solely those of the author(s) and do not necessarily reflect the position of European Publishing. European Publishing accepts no responsibility or liability for any consequences arising from the use of, or reliance on, this content.

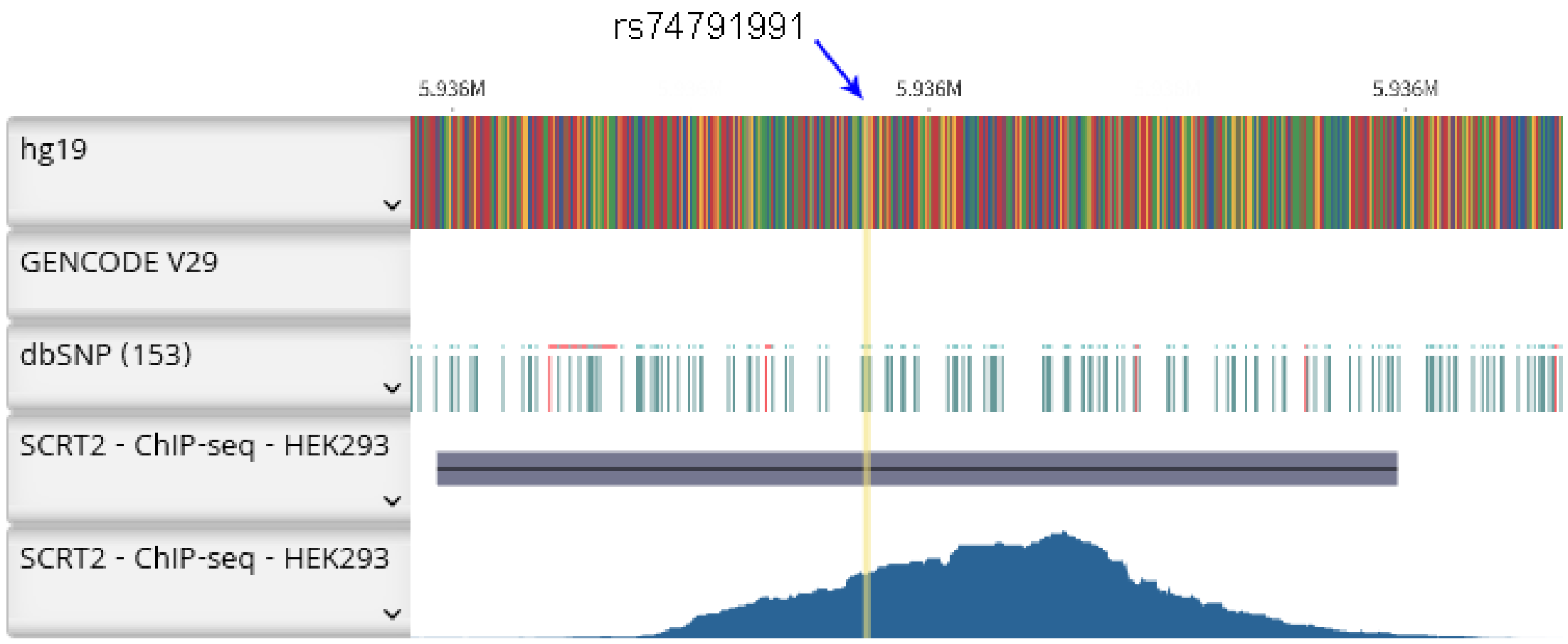

Figure S1. The region of rs74791991 showed a binding activity to the *SCRT2* gene in HEK293 cells. Information were retrieved from the RegulomeDB.

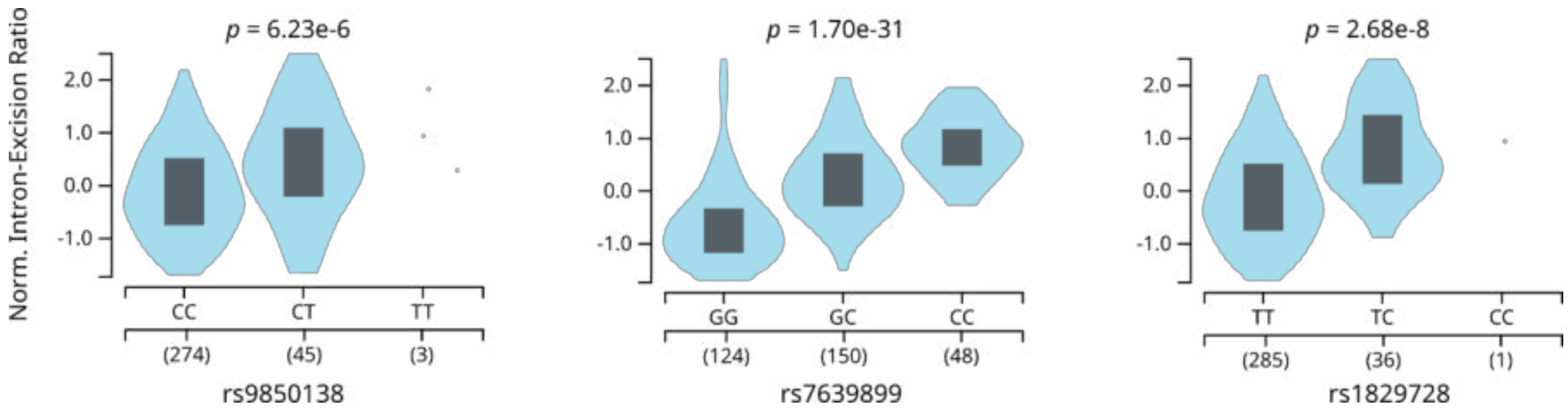

Figure S2. The violin plots of rs9850138, rs7639899, and rs1829728 splicing quantitative trait loci (sQTL) results obtained from the Genotype-Tissue Expression (GTEx) project.

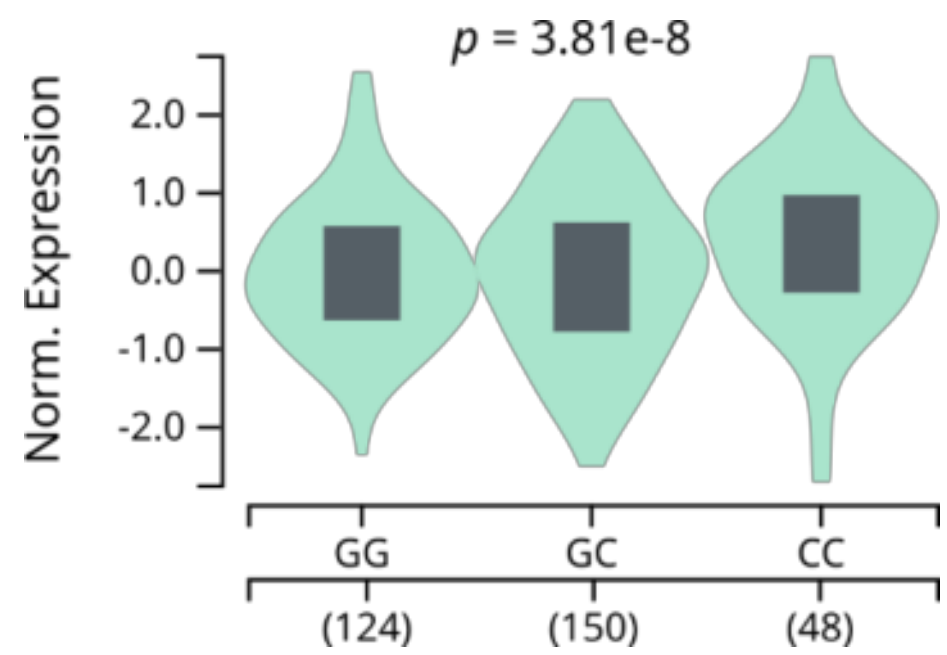

Figure S3. The violin plots of rs7639899 expression quantitative trait loci (mQTL) result obtained from the GTEx project.

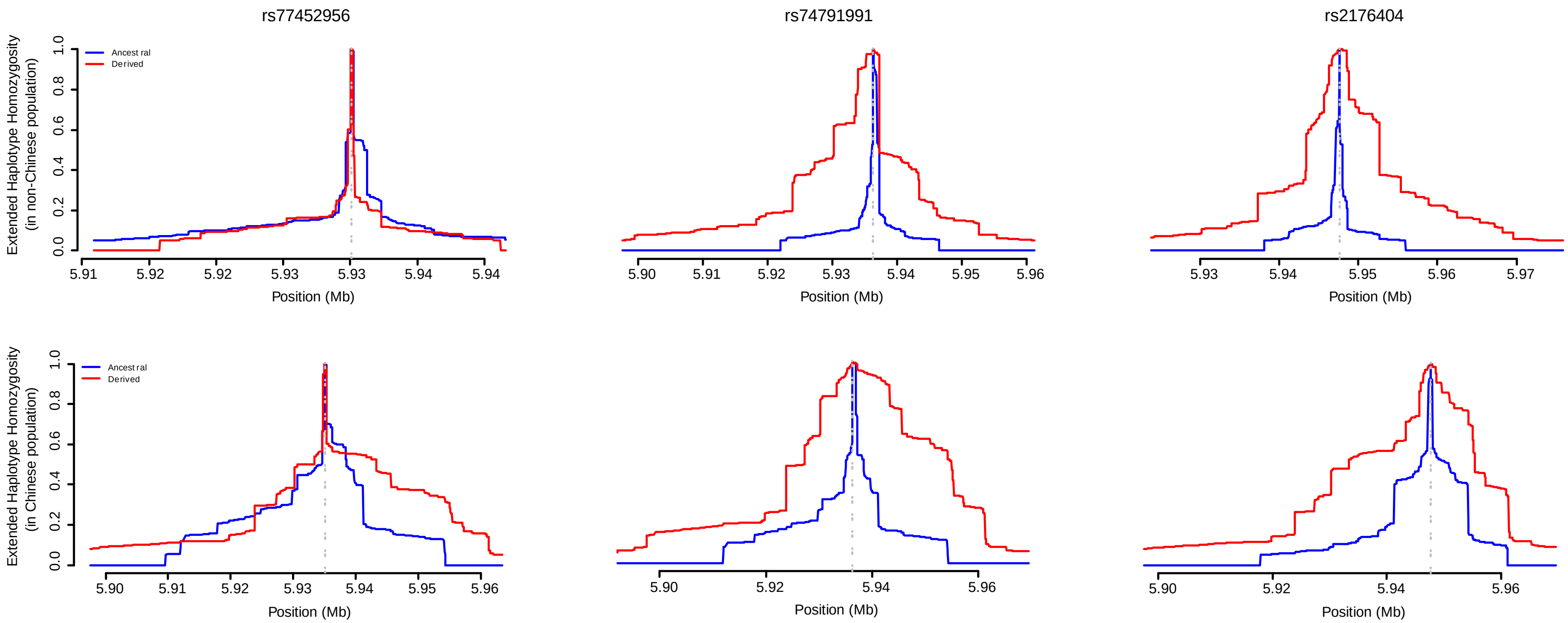

Figure S4. EHH analysis results of rs77452956, rs74791991, and rs2176404, respectively. The lower and upper panels show results from Chinese and non-Chinese populations, respectively.
